# Navigating the evolving landscape of colorectal cancer screening with a practical framework: a comprehensive analysis of existing and emerging technologies for informed decision-making

**DOI:** 10.3389/or.2025.1653617

**Published:** 2025-10-20

**Authors:** Michael Sapienza, Cheryl Davis, Mathieu Boudes

**Affiliations:** ^1^ Colorectal Cancer Alliance, Washington, DC, United States; ^2^ Red Thred Solutions, Nineveh, IN, United States; ^3^ Montsouris Consilium, Montpellier, France

**Keywords:** colorectal cancer, screening, diagnosis, patients, prevention

## Abstract

The colorectal cancer (CRC) screening landscape has rapidly evolved, introducing new technologies alongside established methods. The lack of head-to-head observational studies comparing these diverse options impairs clinicians’ and patients’ ability to make informed choices in CRC screening test selection. This manuscript aims to provide a comprehensive review of existing and emerging CRC screening technologies and develop a practical framework for informed decision-making. We conducted a systematic review of current literature on CRC screening methods, including colonoscopy, fecal immunochemical test (FIT), multi-target stool DNA test (mt-sDNA), the next-generation multi-target stool DNA test, multi-target stool RNA test (mt-sRNA), and blood-based tests. We summarized performance characteristics, adherence rates, follow-up colonoscopy rates, accessibility, and costs for each method. Our review revealed significant variations in test performance, patient adherence, and implementation factors across screening modalities. Blood-based tests showed promise in terms of patient acceptance but currently have lower sensitivity for early-stage cancers with a higher participant adherence when screening navigation is provided. Our review led to the development of a comprehensive framework for evaluating CRC screening options, addressing the critical need for informed decision-making in this area. The framework encompasses five key dimensions: test performance (sensitivity and specificity for CRC and precancerous lesions), patient considerations (invasiveness, preparation, and location preferences), adherence and follow-up (real-world rates and diagnostic colonoscopy completion rates), accessibility and cost (insurance coverage, out-of-pocket expenses, and system integration), and screening interval (recommended frequency and long-term impact). By synthesizing data, the framework enables healthcare providers and patients to navigate the complex landscape of screening options, facilitating personalized recommendations tailored to individual risk factors, preferences, and healthcare system constraints. Future research should validate this framework in diverse clinical settings and update it as new technologies emerge, ensuring continued improvement in CRC screening participation, effectiveness, and outcomes.

## Introduction

CRC remains a significant public health concern in the United States (1) due, in part, to the currently stagnant, and below-target screening rates with traditional modalities. The rapid evolution of CRC screening technologies has introduced a wide array of options, creating a lot of opportunities, ranging from traditional methods like colonoscopy to innovative technologies such as multi-target stool RNA/DNA tests and blood-based tests. While these advancements expand the possibilities for prevention and early detection, they also create complexities for healthcare providers and patients tasked with choosing the most suitable options (1).

This paper proposes a practical framework centered on three essential pillars: data, access, and adherence; to guide informed decision-making in the selection of CRC screening methods. By evaluating existing and emerging tests through these interconnected lenses, the framework enables healthcare providers to balance performance characteristics, accessibility, and real-world adherence. These pillars ensure that decisions are patient-centric, equitable, and aligned with clinical best practices, ultimately improving screening participation and outcomes.

Through a detailed examination of these pillars, this manuscript aims to offer a structured approach to navigating the evolving landscape of CRC screening. This framework not only addresses the challenges posed by diverse screening modalities but also highlights opportunities to enhance screening rates and patient outcomes. It is designed to simplify complex clinical research for the purpose of making key concepts more accessible to a broad range of stakeholders (notably patients and providers). By integrating these concepts throughout the analysis, we aim to empower stakeholders with actionable insights to optimize screening strategies.

## Methodology

To comprehensively evaluate CRC screening technologies and develop an informed decision-making framework, we employed a multi-faceted approach combining systematic literature review, data extraction, and comparative analysis. This methodology aimed to synthesize evidence on screening modalities while considering real-world adherence, accessibility, and performance metrics.

### Literature review and data collection

We conducted a systematic review of peer-reviewed literature, clinical guidelines, regulatory documents, and health system reports published up to March 2025. Our search strategy utilized major medical databases, including PubMed, with a combination of controlled vocabulary and free-text terms related to CRC screening methods, test performance, adherence, accessibility, and cost-effectiveness. Specific keywords were used to capture relevant studies, such as “colonoscopy,” “fecal immunochemical test (FIT),” “multi-target stool DNA test (mt-sDNA),” “multi-target stool RNA test (mt-sRNA),” and “blood-based CRC screening.” These terms were combined using Boolean operators to ensure a comprehensive search. Inclusion criteria included studies published in English that evaluated the sensitivity and specificity of CRC screening tests (colonoscopy, FIT, mt-sDNA, mt-sRNA, and blood-based tests), as well as those reporting adherence rates, follow-up colonoscopy completion, accessibility, cost, and coverage in different healthcare settings. The data extraction was performed by two independent reviewers to ensure consistency and accuracy.

### Development of the evaluation framework

Based on synthesized data, we developed a structured framework encompassing three key pillars: data (test sensitivity, specificity, and clinical performance), access (availability, affordability, and logistical feasibility), and adherence (initial participation and follow-up compliance). Each pillar is thought to provide information through the lens of the patients and their family, the providers and the healthcare systems. The framework is designed to accommodate emerging technologies and evolving evidence.

A draft evaluation framework was presented and debated during a panel discussion at the Screening Dinner, organized by the Colorectal Cancer Alliance in May 2024. Insights from panelists across relevant stakeholder groups were incorporated into the final framework iteration.

### Limitations

Our methodology provides a comprehensive assessment of CRC screening options, though it is not without limitations. This work was designed as a structured narrative review with stakeholder input, rather than a full systematic review. As such, it does not include certain systematic review elements. Existing, approved screening tests benefit from real-world performance data, while newer technologies (those not yet approved or widely available) lack such evidence. Study design heterogeneity and variability in adherence definitions may also introduce variability across studies. Moreover, the absence of large-scale head-to-head comparisons among newer screening modalities necessitates cautious interpretation of indirect comparisons. Finally, our synthesis aimed at providing a practical, accessible framework for decision-making, rather than definitive comparative estimates. Future research should aim to validate and refine this framework across diverse populations and healthcare settings, ideally supported by systematic evidence reviews and decision-analytic modeling.

## Results

The landscape of CRC screening has evolved significantly in recent years, with several new technologies emerging alongside established methods. This analysis focuses on six key screening modalities: colonoscopy, fecal immunochemical test (FIT), multi-target stool DNA tests (mt-sDNA, including Cologuard and Cologuard Plus), multi-target stool RNA test (mt-sRNA, ColoSense), and cell-free DNA blood-based tests (Shield and PREEMPT CRC).

### Performance characteristics

#### Sensitivity and specificity

Evaluation of the performance characteristics of newer tests, particularly sensitivity and specificity, is crucial for understanding their effectiveness in detecting early-stage cancers and precancerous lesions.

Table 1 presents the sensitivity of each screening method for detecting CRC overall and at different stages, as well as advanced precancerous lesions (APL) and advanced adenomas (AA) with definitions that are not the same in all studies and publications. It is worth noting that most of the data was not retrieved from head-to-head studies.

**TABLE 1:** Sensitivity of CRC screening methods. It is important to note that the data presented in Table 1 and the subsequent Tables 2–4 derive from heterogeneous study designs, thresholds, and populations. As such, results are not directly comparable across modalities, and indirect comparisons should be interpreted as hypothesis-generating rather than definitive.

Sensitivity	Colonoscopy	FIT	Cologuard	Cologuard plus	ColoSense	Shield	PREEMPT CRC
Test Type	Visual endoscopy	Hemoglo-bin in stool	mt-sDNA	mt-sDNA	mt-sRNA	Cell-free DNA blood test	(Blood)
CRC overall	95% (2, 3)	79% (4)	92% (5)	94% (6)	94.4% (7)	83% (8)	81.1% (9, 10)
Stage I	75%–80% (11, 12)	75% (4)	90% (5, 13)	87% (6)	100% (7)	65% (55% clinical) (8)	63.5% (9, 10)
Stage II	85%–90% (11, 12)	88% (4)	100% (5)	94% (6)	83% (7)	100% (8)	100% (9, 10)
Stage III	85%–90% (11, 12)	82% (4)	90% (5)	97% (6)	100% (7)	100% (8)	80.5% (9, 10)
Stage IV	>95% (11, 12)	89% (4)	75% (5)	100% (6)	100% (7)	100% (8)	100% (9, 10)
APL/AA	90%–95% (14)	24% (APL) (4)	42% (APL) (5)	43% (APL) (6)	46% (AA) (7)	13.2% (8)	13.7% (AA) (9, 10)
High grade dysplasia	75%–93% (2)	—	69% (5)	75% (6)	65% (HGD or ≥10 adenomas) (7)	22.6% (8)	29% (9, 10)
Sessile serrated	70%–80% (2)	5% (4)	42% (5)	46% (6)	17% (hyperpla-stic and SS ≥ 10 mm combined) (7)	11% in SSL’s greater than 1 cm (8)	—

**TABLE 2:** Specificity of CRC screening methods.

Specificity	All	Negative colonoscopy
Colonoscopy	90% (2, 3)	—
FIT	93% (4)	—
Cologuard	87% (5)	93% (5)
Cologuard Plus	94% (6)	93% (6)
ColoSense	86% (7)	88% (7)
Shield	89.6% (advanced neoplasia) (8)	—
PREEMPT CRC	91.5% (non-advanced colorectal neoplasia) (10)	—

Colonoscopy has long been considered the test with the highest sensitivity for CRC overall and is the benchmark against which other tests are compared, though the next-generation mt-sDNA test and the mt-sRNA test has been shown to have similar cancer sensitivity as colonoscopy (2). Stool-based tests offer varying levels of sensitivity for CRC and APL/AA, with multi-target stool DNA (mt-sDNA) and multi-target stool RNA (mt-sRNA) tests generally outperforming FIT, especially for early-stage cancers and advanced precancerous lesions (5–7). Both the mt-sDNA tests and the mt-sRNA test have proven to have higher sensitivity as compared to FIT in head-to-head pivotal trials (6, 7). mt-sDNA tests are more likely to detect sessile serrated lesions (so-called “flat lesions”) than FIT or mt-sRNA. As of March 2025, there is no head-to-head comparison between mt-sDNA and mt-sRNA. The performance for stool-based tests in terms of CRC sensitivity ranged from 74%–94%, while the performance for CRC sensitivity in blood-based test was 83% (8, 10). It is worth noting that recent publications have suggested wide variability in FIT performance depending on test used (15).

Specificity is generally high across all testing modalities, with most methods demonstrating specificity at or above 90% except for mt-sRNA and mt-DNA.

It's important to note that lower specificity may lead to more follow-up colonoscopies, which can impact healthcare resources and patient experience. However, the high specificity across all testing modalities suggests that false positives are relatively rare (4–14, 5% (6, 7, 16)), which is crucial for maintaining patient trust and minimizing unnecessary procedures.

#### Testing intervals and adherence rates

The interval and adherence rates (Table 3) for different CRC screening methods vary significantly. Colonoscopy has the longest recommended interval at 10 years (18), with adherence rates of about 30% in real-world settings. Fecal immunochemical test (FIT) has an annual interval, with adherence rates of 35% without navigation and 41.5% in real-world and study settings. Cologuard, a multi-target stool DNA test, has a recommended interval of 3 years (with a range of 1–3 years), and shows adherence rates of 71.3% overall with commercial insurance at 72.3%, Medicare Advantage at 70.2%, Medicare at 69.9%, and Medicaid at 52% (19).

**TABLE 3:** Interval between testing and adherence of the different CRC screening methods.

Screening methods	Interval (years)	Adherence (%)
Colonoscopy	10 (23)	about 30% (24)
FIT	1 (23)	35% (w/o navigation) 41,5% (w navigation) (real-world and study) (20, 25–32)
Cologuard	3 (1–3, 27, 28)	71% (real-world) (20, 29)
Cologuard Plus	3 (anticipated)	anticipated as the same as Cologuard
ColoSense	3 (anticipated)	78% (study) (7)
Shield	1–3 (to be determined)	96% (real-world) (30)
PREEMPT CRC	3 (31)	96% (study) (32)

Cologuard Plus, the next-generation, will maintain a 3-year interval and is expected to mirror that of Cologuard, since ordering processes and patient experience will not change with study adherence reported at 71% (20). ColoSense, a multi-target stool RNA test, also has an anticipated 3-year interval and does not yet have real world adherence. It demonstrated an 78% adherence rate in study settings. The blood-based test, Shield, has an adherence of 96% based on real world clinical usage and integrating a blood-based test into CRC screening discussion leads to a twofold increase of the screening rates (21), with Shield’s interval to be determined (estimated 1–3 years) while PREEMPT CRC’s proposed at 3 years. It is worth noting that adherence rates for newer tests are often based on study data and may differ in real-world applications. A recent systematic literature review across 36 million patients undergoing routine USPSTF recommended blood-based screening tests, adherence rates ranged from 34%–68% indicating that outside of clinical trials, real-world adherence to recommended blood-based screening may be suboptimal (22).

#### Follow up colonoscopy

Colonoscopy follow-up, access, and costs are critical factors in evaluating the effectiveness of CRC screening programs. According to the data presented in Table 4, follow-up colonoscopy rates vary significantly across different screening modalities. For FIT, follow-up rates range from 47% to 83%, highlighting the importance of robust tracking and reminder systems (33). Cologuard demonstrates higher follow-up rates of 71.5%–84.9% in real-world settings. While neither ColoSense nor Shield have conducted studies with endpoints that look at follow up colonoscopy adherence, ColoSense showed an 88% follow-up rate in study settings and Shield had a rate of 44% in a real world, claims analysis for individual with a colonoscopy within 6 months of the positive test (34, 35), emphasizing the need for improved follow-up strategies for blood-based tests.

**TABLE 4:** Follow-up colonoscopy, access and cost of the different CRC screening methods (7, 8, 17–25, 33–36).

Screening methods	Follow up colonoscopy	Access	Cost
Colonoscopy	N/A	Medicare.gov	$2.750 (avg. Cash price)
FIT	47%–83% (33)	Widely available/covered	$18 - $21
Cologuard	71.5% - 84,9% (real-world) (38, 39)	Widely available/covered	$508 (Medicare) (inc. Screening navigation)
Cologuard Plus	—	Not currently available	$592 (Medicare)
ColoSense	88% 74% combined test and follow up (study) (34)	Not currently guideline-recommended but is FDA-approved to screen for CRC, advanced adenomas, and sessile serrated lesions, in average-risk individuals over the age of 45	$508 (Medicare)
Shield	44% (real world) (35)	available under the CRC screening National Coverage Determinations (NCDs)	$1,495 (Covered by Medicare; $0 out of pocket cost for Medicare Part B patients)
PREEMPT CRC	—	Not currently available	—

#### Access and cost

Access to screening tests varies, with colonoscopy, FIT and Cologuard being widely available and covered by most insurance plans. Shield is available under the CRC screening National Coverage Determinations (NCDs). However, newer tests like ColoSense are not currently guideline-recommended but is FDA-approved to screen for CRC, advanced adenomas, and sessile serrated lesions, in average-risk individuals over the age of 45. Cost considerations play a significant role in screening implementation. Colonoscopy has the highest cost at an average cash price of $2,750, which may present a barrier for uninsured or underinsured individuals. FIT is the most affordable option, ranging from $18 to $21 with an estimation of $153 per screening cycle when including the patient support costs (37). Cologuard and ColoSense have a Medicare reimbursement rate of $508, and $592 for Cologuard Plus, positioning them as mid-range options that also have screening navigation programs built into the price of the tests. Further, there is no charge for Cologuard or ColoSense until resulted, meaning the manufacturers bear the burden of ensuring a kit’s return, which is not the case with most FIT kits. The announced cash price for Shield is $1,495, which may limit its accessibility without adequate insurance coverage. It is available with no out-pocket costs for patients eligible for Medicare Part B. There are other considerations as well. As newer tests enter the market, their cost and coverage will be crucial factors in determining their accessibility and impact on overall screening rates and health system resources.

### Factors influencing test choice

The choice of CRC screening test is influenced by various factors related to test performance, characteristics, and contextual elements. Understanding these factors is crucial for improving screening rates and outcomes.

#### Test performance and characteristics

Test performance is a key consideration in CRC screening. Colonoscopy, sometimes referred to as the gold standard, has high sensitivity (95%) for detecting CRC and advanced precancerous lesions. Stool-based tests show sensitivity for CRC detection at 79% (FIT), 92% (mt-sDNA) and up to 94% for ColoSense and Cologuard Plus, though lower sensitivity for advanced precancerous lesions (precancerous lesion performance is highly heterogeneous, and was not systematically assessed due to the lack of available data and should be interpreted with caution). Emerging blood-based tests, while promising, currently show similar sensitivity for advanced adenomas compared to stool-based tests (40). Test characteristics significantly impact patient preferences. Stool-based tests are often preferred over colonoscopy due to their non-invasive nature, convenience, and ability to be completed at home. A national survey found that 65.4% of respondents preferred mt-sDNA tests over colonoscopy, and 61% preferred FIT/gFOBT over colonoscopy (41). Handling stool samples may be a barrier for some patients, making blood-based tests a potential alternative for those patients who would not otherwise get screened, however the patients need to travel to a venipuncture site and some patients may fear needles. Test interval is another important characteristic, with patients generally preferring longer intervals between screenings (41).

#### Contextual factors

Contextual factors such as travel distance, insurance coverage, health system test, geography, access availability of colonoscopy impact screening decisions. A study in rural Southern Illinois found that long-distance travel for care was normalized but not necessarily preferable for patients. Providers identified distance-related challenges specific to CRC screening, including transportation issues and the need for patients to take time off work (42). Insurance coverage also influences screening choices. Among adults aged 45–49 years, those with Medicare or Medicaid were more likely to prefer colonoscopy (62.8%) compared to those with private insurance (54.8%) or no insurance (46.7%) (41). Uninsured individuals are more likely to prefer stool-based tests over colonoscopy due to cost considerations.

Geographic location plays a role, with rural residents facing unique barriers to CRC screening. In rural Southern Illinois, providers reported that distance to care remains a significant challenge to increasing CRC screening and contributes to disparities in rural communities (42–44). They suggested various solutions to reduce distance and transportation barriers, such as mobile screening units and telemedicine options for pre-screening consultations.

#### Patient preferences

Patient preferences are shaped by multiple factors. Convenience and ease of use are primary considerations, with many patients citing forgetfulness or procrastination as reasons for not completing stool-based tests (45, 46). A study on pharmacy-based CRC screening programs found that 93% of participants preferred completing the test at home, and 85% valued the ability to drop off the completed test at a convenient location. Perceived accuracy also influences choice, with some patients believing blood tests to be more accurate than stool tests, despite lacking information on actual test characteristics. In a qualitative study, patients were receptive about completing a blood test for CRC screening, citing simplicity, ease, convenience, and high perceived accuracy (47, 48). Cultural factors can impact screening preferences. A study focusing on Latino patients found varying levels of awareness about FIT testing, with some expressing doubts about its efficacy compared to colonoscopy (49). This underscores the need for culturally tailored education and outreach efforts.

#### Healthcare provider preferences

Healthcare provider preferences are influenced by factors such as test accuracy, patient compliance, and system integration. A survey of 1,281 primary care providers found that 82.9% rated colonoscopy as very effective for patients aged 50-74, compared to 59.6% for FIT (50). Moreover, 26.3% rated colonoscopy as more effective than FIT, despite some modeling studies suggesting comparable effectiveness. Providers express concerns about patient adherence to different screening modalities. The same survey found that providers recommended colonoscopy every 10 years for 77.9% of patients aged 50-74, while 92.4% recommended FIT annually. System integration is crucial, with providers favoring options that are easy to implement within their healthcare systems. The ability to integrate screening programs with electronic health records and existing workflows is an important consideration for many healthcare organizations (51).

#### Education and communication

Education and communication play vital roles in test choice and screening adherence. Both patients and providers express a need for clear, comprehensive information about different screening options (52–54). A study on Latino patients’ perceptions of FIT testing found that awareness levels varied based on prior screening experiences, highlighting the need for targeted education efforts, especially for underserved populations (49). Providers may benefit from education about the comparative effectiveness of different screening modalities. The survey of primary care providers revealed discrepancies between provider perceptions and evidence from modeling studies regarding the effectiveness of FIT compared to colonoscopy (50). Addressing these knowledge gaps could influence provider recommendations and, subsequently, patient choices. Effective communication strategies and decision aids can help patients make informed choices aligned with their preferences and values. A study on older adults found that a targeted patient decision aid influenced screening preferences, particularly among those in poorer health states (55). This suggests that decision aids can be valuable tools for facilitating shared decision-making about CRC screening.

#### Emerging technologies and future directions

As technology advances, new screening options are emerging that may further influence test choices. Artificial intelligence (AI) is being explored to enhance the accuracy of existing screening methods. For instance, AI-assisted colonoscopy has shown promise in improving adenoma detection rates (56, 57). These emerging technologies may address some of the current barriers to screening, such as the invasiveness of colonoscopy or the discomfort associated with stool-based tests. However, their implementation will require careful consideration of factors such as cost-effectiveness, accessibility, and integration into existing healthcare systems (58, 59).

#### Policy implications

Policies that address barriers to screening, such as lack of insurance coverage or limited access to healthcare facilities, can significantly impact screening rates and test choices. For instance, the Affordable Care Act’s requirement for insurance plans to cover preventive services, including CRC screening, has been associated with increased screening rates (60).

The choice of CRC screening test is influenced by a complex interplay of factors related to test performance, characteristics, context, and stakeholder preferences. Understanding and addressing these multifaceted influences is key to optimizing CRC screening strategies and outcomes. Future efforts to increase screening rates should focus on addressing barriers related to access, education, and communication, while considering the diverse needs and preferences of different patient populations. As new technologies emerge and our understanding of CRC screening evolves, it will be crucial to continually reassess and adapt screening strategies. This may involve integrating new screening modalities, refining risk-stratification approaches, or developing more personalized screening recommendations based on individual risk factors and preferences through early detection and prevention. By carefully considering the various factors that influence screening test choices and working to optimize these choices for different populations and contexts, we can make significant strides towards this important public health goal.

### Modeling studies to inform CRC screening policies

Simulation modeling has emerged as a valuable tool in evaluating CRC screening strategies, offering insights into the complex interplay of factors that influence screening effectiveness and cost-efficiency. These models provide decision-makers with essential information to guide policy and healthcare system choices in the absence of feasible large-scale clinical trials (61, 62). However, it is crucial to understand both the strengths and limitations of these modeling studies in the context of CRC screening methods.

Modeling studies have been instrumental in comparing various CRC screening modalities, assessing their cost-effectiveness, and determining the potential impact of different screening strategies on population health outcomes (63). These studies have consistently shown that CRC screening, regardless of the method, is cost-effective compared to no screening (64, 65). They have also provided valuable insights into the relative effectiveness of different screening methods, such as colonoscopy, sigmoidoscopy, and stool-based tests, under various assumptions and scenarios (62, 63). One of the key strengths of modeling studies is their ability to simulate long-term outcomes and evaluate the impact of screening strategies over extended periods, which would be impractical or impossible in real-world trials (61, 63). This capability allows researchers to estimate the potential reduction in CRC incidence and mortality rates associated with different screening approaches, helping to inform policy decisions and resource allocation (62). Moreover, modeling studies can incorporate a wide range of variables, including screening and follow-up colonoscopy adherence rates, test characteristics, and cost parameters, to provide a comprehensive assessment of screening strategies. This flexibility allows for the evaluation of various “what-if” scenarios, helping decision-makers understand the potential impact of changes in screening policies or technologies (61, 65).

Despite their utility, modeling studies have limitations that must be considered when interpreting their results. Firstly, the validity and reliability of these models heavily depend on the quality and accuracy of the input data and assumptions used (62, 66). Many studies fail to report full model validation, with only 58% of reviewed studies conducting any validation at all (63). This lack of thorough validation can lead to uncertainties in the model’s predictions and potentially misleading conclusions. Secondly, while modeling studies can simulate various scenarios, they cannot capture the full complexity of real-world implementation challenges. Factors such as patient preferences, real-world adherence to initial and longitudinal screening, healthcare provider behavior, and system-level barriers to screening implementation are often difficult to accurately represent in models (61). As a result, the actual impact of a screening strategy may differ from model predictions when implemented in practice. Furthermore, modeling studies typically focus on average-risk populations and may not adequately address the needs of specific subgroups or individuals with varying risk profiles. This limitation can lead to oversimplified recommendations that may not be optimal for all segments of the population (62, 65). Lastly, while modeling studies can provide valuable evidence for decision-making, they often fall short in reporting their actual impact on health system or policy decisions. A systematic review found that while 91% of models were developed to address specific health system or policy questions, only 12% reported on the model’s impact on these decisions (63). This gap highlights the need for better integration of modeling results into the decision-making process and improved communication between researchers and policymakers.

Modeling studies offer valuable insights into the potential effectiveness and cost-efficiency of CRC screening methods. However, their limitations, including validation concerns, real-world implementation challenges, and the gap between model development and policy impact, must be carefully considered. To maximize the utility of these models in informing CRC screening policies, there is a need for more rigorous validation processes, improved representation of real-world complexities, and better mechanisms for translating model findings into practice.

### A practical framework for evaluating CRC testing options

**Table:** 

Data Focuses on the test’s sensitivity, specificity, and clinical performance	Key considerations • Sensitivity and specificity for detecting CRC and precancerous lesions • Stage-specific performance (e.g., early-stage cancers vs. advanced lesions) • Comparative data across screening modalities (see figures above)			
	**Stakeholder implications**	**Patients and family** Clear explanations of test accuracy to empower informed choices	**Healthcare systems** Data-driven resource allocation for maximizing impact	**Providers** Confidence in recommending evidence-based options
Access Encompasses the availability, affordability, and logistical feasibility of screening methods	Key considerations • Insurance coverage and out-of-pocket costs • Financial assistance programs for under and uninsured • Geographic accessibility, including rural and underserved areas • Integration with healthcare systems (e.g., electronic health records, workflows)			
	**Stakeholder implications**	**Patients and family** Minimizing barriers such as transportation and cost	**Healthcare systems** Strategies to enhance availability and reduce inequities	**Providers** Ensuring compatibility with existing clinical workflows
Adherence Addresses initial participation and follow-up compliance	Key considerations • Real-world adherence rates for initial testing, follow up colonoscopy, and repeat testing • Evidence-based strategies, such as built-in navigation programs or personalized follow-up reminders • Patient-centered education campaigns tailored to literacy levels and cultural preferences • Implementation of digital reminder systems (including SMS or app-based notifications) • Patient preferences • Collaboration between healthcare systems and community organizations can address barriers to follow-up, particularly in underserved populations			
	**Stakeholder implications**	**Patients and family** Tailored communication to encourage follow-through	**Healthcare systems** Design and implementation of robust adherence programs	**Providers** Navigation programs and simplified protocols to enhance patient compliance

## Discussion

### Framework for evaluating CRC screening options

The proposed framework evaluates three primary attributes: data strength, patient adherence, and access. Given the heterogeneity of study designs and outcome definitions across screening modalities, caution is warranted when interpreting indirect comparisons. These data provide a useful overview of trends but should not be considered as head-to-head evidence. While no single attribute dominates, the effort required to address each varies. If one were to prioritize these attributes, performance data would be a logical starting point. Understanding the sensitivity of tests for detecting polyps, stage I and II cancers, and advanced lesions is essential for maximizing mortality prevention. Currently, the majority of data focuses on sensitivity and specificity for CRC. Decisions based solely on data are impractical. Tests must meet minimum sensitivity and specificity thresholds; however, even high-performing tests lose utility if patients do not adhere. Adherence encompasses not only initial screening but also follow-up. For example, blood-based tests may reduce barriers to initial implementation but may disproportionately reach individuals who are less likely to complete necessary follow-up colonoscopies. This demonstrates the importance of evaluating adherence holistically.

### Challenges and opportunities in access and capacity

Access-related factors, such as colonoscopy availability and capacity, play a pivotal role in decision-making. Colonoscopy capacity must account for follow-up needs arising from other screening modalities. While the ability to offer choice is beneficial, effective population management necessitates clear protocols. FIT testing, for instance, can be paired with outreach strategies, such as phone or text follow-ups, to increase adherence. However, significant barriers remain, especially for populations with limited access to colonoscopy.

### Perspectives on decision-making

Decision-making priorities in healthcare vary significantly among different stakeholders. Patients typically prioritize practical considerations such as time away from work, transportation issues, out-of-pocket expenses, and insurance coverage when making healthcare decisions. Healthcare providers, on the other hand, often emphasize the practicality of tests, adhering to the principle that “*The best test is the one that gets done*” (67). This mindset may lead them to favor non-invasive options like FIT stool-based tests or blood-based tests in certain situations due to their accessibility, although concerns exist about newer options that may have lower sensitivity for detecting pre-neoplastic, high-risk lesions, or early cancers. Health systems and public health entities face their own set of challenges, primarily driven by logistical and financial constraints. These organizations, particularly public health programs, often prioritize cost-effective, non-colonoscopy options such as stool-based tests for outreach initiatives, especially when targeting populations that are less likely to engage with traditional screening methods. Moving forward, greater collaboration among stakeholders is needed to refine screening strategies and improve both adherence and outcomes.

### Innovation’s limitations in detecting precancerous lesions

An important limitation of current CRC screening modalities, particularly non-colonoscopy tests, is their variable and often suboptimal ability to detect precancerous lesions such as advanced adenomas and sessile serrated polyps. While colonoscopy remains the reference standard for identifying and removing these precursor lesions, stool- and blood-based tests have demonstrated significantly lower sensitivity. This shortcoming has major implications for prevention at the population level, as failure to reliably detect precancerous states undermines the long-term effectiveness of screening programs. Addressing this gap represents a key research priority, with innovation urgently needed to improve early lesion detection and thereby enhance the preventive potential of CRC screening.

Although this framework was developed with a focus on the U.S. context, future research should explore its application and adaptation in international settings, including Europe, Asia, and LMICs, where health system structures, access barriers, and patient preferences may differ substantially.

## Conclusion

The rapid evolution of CRC screening modalities highlights the need for a structured approach to navigate the complexities of decision-making. The proposed framework, grounded in the core principles of data, access, and adherence, serves as a robust model for assessing both established and novel CRC screening methods. By methodically evaluating performance metrics, accessibility barriers, and adherence challenges, this framework aims to foster screening strategies that are not only clinically effective but also equitable and centered on patient needs.

Each of the three pillars plays a unique and vital role in optimizing screening outcomes. The pillar of “Data” provides the foundational basis for clinical confidence by prioritizing accuracy and reliability in detecting CRC and its precursors. “Access” ensures that screening options are made equitably available, addressing systemic barriers and resource disparities that could otherwise limit the reach of screening programs. Finally, the “Adherence” pillar targets one of the most critical aspects of effective screening programs: the consistent participation of patients, from initial testing to necessary follow-up procedures. These pillars function synergistically, empowering patients, healthcare providers, and health systems alike to make evidence-informed decisions that align with public health priorities and individual needs.

By integrating this comprehensive framework, stakeholders have the potential to significantly enhance CRC screening strategies, addressing gaps in access and adherence while promoting equity across diverse populations. Furthermore, the implementation of this framework invites opportunities for iterative improvement. Future research efforts should aim to validate the framework’s utility in varied clinical settings and among diverse populations, while policy initiatives should strive to operationalize its principles within healthcare systems. Engaging all relevant stakeholders in this process (patients, providers, payers, and policymakers) will be crucial to sustaining progress. As the landscape of CRC screening continues to evolve, this framework provides a dynamic foundation for advancing prevention and early detection, ultimately reducing the global burden of CRC. Moreover, the scientific community should explore ways to assigning weights and testing the framework in a formal decision-analytic or scoring model to assess its applicability in real-world clinical decision-making.
